# Antiepileptic Drug Adherence in Patients with Epilepsy in Saudi Arabia: A Multicentre Cross-Sectional Study

**DOI:** 10.3390/healthcare14152302

**Published:** 2026-07-30

**Authors:** Abdullah A. Alshehri, Wael Y. Khawagi, Khawlah I. Alshahrani, Razan A. Alshehri, Ghadeer A. Alhawsawi, Deemah H. Altowairqi, Bayan A. Alqarni, Norah M. Alotibi, Rawan S. Alshahrani, Wejdan A. Alzahrani, Waleed M. Altowayan, Saber Chebel, Ali F. Alnumair, Yahya H. Alzahrani, Ahmed Ibrahim Fathelrahman

**Affiliations:** 1 Department of Clinical Pharmacy, College of Pharmacy, Taif University, Taif 21944, Saudi Arabia; w.khawagi@tu.edu.sa (W.Y.K.);; 2 King Abdullah bin Abdulaziz University Hospital, Riyadh 11564, Saudi Arabia; 3 College of Pharmacy, Taif University, Taif 21944, Saudi Arabia; 4 Department of Pharmacy, Dr. Soliman Fakeeh Hospital, Jeddah 21461, Saudi Arabia; 5 Department of Pharmacognosy, College of Pharmacy, Najran University, Najran 61441, Saudi Arabia; 6 Department of Pharmacy Practice, College of Pharmacy, Qassim University, Buraydah 52571, Saudi Arabia; 7 Department of Neurology, Dr. Sulaiman AlHabib Hospital, Buraidah 52381, Saudi Arabia; 8 Pharmacy Department, Proceed Healthcare Logistics, Al-Dawaa Medical Services Company, Riyadh 31952, Saudi Arabia; 9 Department of Neurology, King Abdulaziz Specialist Hospital, Taif 26521, Saudi Arabia

**Keywords:** epilepsy, antiepileptic drugs, medication adherence, seizure control, Saudi Arabia, cross-sectional study

## Abstract

**Background**: Medication adherence is essential for optimal seizure control in epilepsy. However, non-adherence to antiepileptic drugs (AEDs) remains common and contributes to poor clinical outcomes. Evidence on adherence patterns and determinants in Saudi Arabia is limited. This study aimed to evaluate AED adherence and identify factors associated with adherence among patients with epilepsy in Saudi Arabia. **Methods**: A multicentre cross-sectional study was conducted among patients with epilepsy attending outpatient clinics across multiple regions in Saudi Arabia. Data were collected between January 2023 and June 2025. Medication adherence was assessed using the self-reported 10-item Medication Adherence Rating Scale (MARS). Adherence was categorized as low (0–5), moderate (6–7), or high (8–10). Descriptive statistics summarized participant characteristics. Logistic regression was used to identify factors associated with high adherence, reported as adjusted odds ratios (aORs) with 95% confidence intervals. All analyses were performed using Stata. **Results**: A total of 534 participants were included. Nearly half reported a seizure during the previous six months (46.4%). High adherence was observed in 58.2% of participants while moderate adherence was reported by 27.5%, and 14.2% had low adherence. The most common adherence barrier was forgetfulness (57.3%). Fear of medication side effects was reported by 27.9% of participants. Seizure occurrence was associated with lower adherence (aOR 0.39, 95% CI 0.26–0.59). Each additional adherence barrier reduced the odds of high adherence (aOR 0.71, 95% CI 0.63–0.80). More reported side effects were also associated with lower adherence (aOR 0.92, 95% CI 0.87–0.97). Participants from the Eastern (aOR 5.06, 95% CI 1.14–22.35) and Central regions (aOR 5.36, 95% CI 1.48–19.38) showed higher adherence than those from the Southern region. **Conclusions**: Medication adherence among patients with epilepsy in Saudi Arabia remains suboptimal. Modifiable barriers, particularly forgetfulness and treatment side effects, should be addressed. Patient-centred adherence interventions may improve long-term seizure control and treatment outcomes. These findings should be interpreted considering the cross-sectional study design and the use of a self-reported adherence measure.

## 1. Introduction

Epilepsy is one of the most common chronic neurological disorders worldwide. It affects more than 50 million people and contributes substantially to disability, reduced quality of life, and premature mortality [1,2,3,4]. Effective seizure control depends predominantly on sustained adherence to antiepileptic drug (AED) therapy [5,6,7]. Adequate adherence to prescribed AED regimens can achieve seizure control in up to two-thirds of patients [8]. However, suboptimal adherence remains a major challenge in routine clinical practice [7,8].

Medication non-adherence in epilepsy is widely recognized as a multifactorial problem influenced by patient-related, therapy-related, socioeconomic, and health system factors [6,9,10]. These may include treatment complexity, adverse drug effects, forgetfulness, misconceptions about long-term therapy, psychological comorbidities, medication cost, and accessibility of healthcare services. Poor adherence has been consistently associated with increased seizure recurrence, emergency department visits, hospitalisations, injury risk, reduced functional outcomes, and increased healthcare utilization, ultimately imposing significant clinical and economic burdens on healthcare systems [7,8,11].

Evidence indicates that adherence to AED therapy varies considerably across countries and healthcare settings due to differences in healthcare infrastructure, patient education, cultural perceptions of chronic illness, and continuity of care [12,13,14]. In low- and middle-income settings and populations facing healthcare access barriers, lower adherence rates are frequently reported. Understanding context-specific determinants of adherence is therefore essential for developing targeted interventions aimed at improving treatment outcomes.

In Saudi Arabia, epilepsy care has expanded substantially in recent years through improved access to specialist services and the wider availability of antiepileptic medications [15,16]. Despite these improvements, epilepsy continues to be associated with social stigma, variable health literacy, and challenges related to long-term medication adherence [17,18,19]. Previous Saudi studies evaluating antiepileptic drug adherence have largely been limited to single healthcare institutions or specific geographic regions, limiting the generalizability of their findings and providing only a partial understanding of adherence patterns nationwide [20,21,22]. Moreover, evidence regarding factors associated with adherence across different healthcare settings remains limited.

Medication adherence can be assessed using both objective methods, such as prescription refill records, pill counts, and electronic medication monitoring, and validated self-report instruments. While objective measures may provide more direct estimates of adherence, self-report tools are practical for large multicentre studies and can capture patient-reported barriers to medication adherence [23]. Therefore, this multicentre cross-sectional study was conducted to evaluate medication adherence among patients with epilepsy across multiple healthcare institutions in different regions of Saudi Arabia and to identify demographic, clinical, and treatment-related factors associated with adherence using a validated self-report instrument. In addition, this study explored patient-reported barriers and treatment experiences that may influence medication-taking behaviour and seizure control. By generating multicentre real-world evidence, this study seeks to inform healthcare planning and support the development of targeted, patient-centred strategies to optimize treatment adherence and improve epilepsy outcomes.

## 2. Methods

This study followed the Strengthening the Reporting of Observational Studies in Epidemiology (STROBE) guidelines for cross-sectional studies.

### 2.1. Study Design and Setting

A multicentre, descriptive cross-sectional study was conducted to assess adherence to AED therapy and its associated determinants among patients with epilepsy in Saudi Arabia. Participants were recruited from adult neurology outpatient clinics at five secondary and tertiary healthcare institutions located in four cities (Jeddah, Dammam, Taif, and Al-Qassim Buraidah) between January 2023 and June 2025. The geographical region reported in the analysis refers to the participants’ region of residence rather than the location of the recruiting hospital. Therefore, participants from all major regions of Saudi Arabia were represented in the study, regardless of the recruiting centre. A summary of the participating centres, recruitment by centre, and the distribution of participants according to their region of residence is provided in Supplementary Table S1.

### 2.2. Participants

Patients with a neurologist-confirmed diagnosis of epilepsy who had been receiving AED therapy for at least six months were eligible for inclusion. Participants were recruited from adult neurology outpatient clinics, which provide care for patients from 16 years of age. Participants were required to complete the study questionnaire independently or with caregiver assistance when appropriate.

### 2.3. Questionnaire Development and Structure

A structured, self-administered questionnaire was developed to assess AED adherence and its associated determinants among patients with epilepsy. The questionnaire was informed by published research on medication adherence in epilepsy and associated barriers and refined through expert consultation [15,17,20]. The final survey comprised 32 items covering sociodemographic characteristics, clinical and treatment-related information, medication adherence, and potential determinants of adherence. The questionnaire required approximately 7 min to complete.

The questionnaire was initially developed in English and translated into Arabic using forward–backward translation procedures to ensure linguistic and conceptual equivalence. Content and face validity were evaluated by a multidisciplinary panel comprising clinical pharmacy academics and neurology specialists.

### 2.4. Piloting the Questionnaire

Pilot testing was conducted among 15 patients with epilepsy to assess clarity, comprehension, and feasibility prior to full-scale administration. Minor refinements were implemented following participant feedback, and responses obtained during pilot testing were excluded from the final analysis.

### 2.5. Measures

The primary outcome was adherence to AED therapy. Medication adherence was assessed using the validated 10-item Medication Adherence Rating Scale (MARS), which evaluates behavioural and attitudinal aspects of medication-taking behaviour over the preceding week [24]. Responses were recorded in dichotomous (Yes/No) format and summed to produce total scores ranging from 0 to 10, with higher scores indicating better adherence. For descriptive analyses, adherence was categorized into three predefined levels: low (0–5), moderate (6–7), and high (8–10). These categories were used to facilitate the interpretation of adherence levels and comparison across participant subgroups. Explanatory variables included sociodemographic characteristics (age group, sex, nationality, marital status, education level, employment status, income, and geographic region), clinical characteristics (epilepsy type, age at diagnosis, seizure occurrence within the previous six months, family history of epilepsy, and duration of therapy), and treatment-related factors, including regimen type (monotherapy or polytherapy), healthcare setting (public versus private hospitals), and the type and number of prescribed AEDs. Additional self-reported measures assessed adherence barriers and medication-related adverse effects. Composite variables representing the number of reported adherence barriers, the number of adverse effects, and the number of medications were generated and included as continuous predictors in subsequent analyses.

### 2.6. Data Collection

Data were collected using a structured questionnaire administered primarily through an anonymous electronic survey hosted on a secure online platform (Google Forms). Eligible participants attending neurology outpatient clinics at the five participating hospitals were invited to participate by their treating physicians who provided either an electronic survey link or a paper-based questionnaire. For participant convenience, paper questionnaires were offered to individuals who preferred in-person completion, and responses were subsequently entered into the electronic database by trained research personnel. Recruitment from multiple secondary and tertiary hospitals across different regions of Saudi Arabia was intended to enhance the diversity of the study population and improve the representativeness of patients receiving specialist epilepsy care.

### 2.7. Sample Size Calculation

The minimum required sample size was estimated using the Raosoft sample size calculator, assuming a 50% expected prevalence of medication adherence, a 95% confidence level, and a 5% margin of error, resulting in a target sample size of 384 participants.

### 2.8. Statistical Analysis

Data completeness was assessed before analysis. Fifty-two respondents were excluded because they did not meet the eligibility criteria. Missing data were minimal (one missing value for epilepsy type), and analyses were performed using available-case analysis without imputation. Descriptive statistics were used to summarize participant characteristics, treatment profiles, and adherence outcomes. Continuous variables were presented as means and standard deviations, while categorical variables were reported as frequencies and percentages. Univariate logistic regression analyses were performed to estimate crude associations between each independent variable and high adherence. Variables with a *p*-value < 0.20 in univariate analysis were considered eligible for inclusion in the multivariable model. A multivariable logistic regression model was then constructed using the enter method to estimate adjusted odds ratios (aORs) with corresponding 95% confidence intervals (CIs). Categorical variables were entered as indicator (dummy) variables. Continuous variables were modelled as linear predictors after assessment of linearity in the logit. Model fit was assessed using the likelihood ratio test and pseudo R^2^ statistics. Statistical significance was defined as a two-sided *p*-value < 0.05. All analyses were performed using Stata version 16 (StataCorp, College Station, TX, USA).

### 2.9. Ethical Considerations

Ethical approval for this study was obtained from the Institutional Review Board (IRB) of Taif University, Saudi Arabia (Approval No. 44-099; dated 27 November 2022) and the Qassim Regional Research Ethics Committee (Ref: 607/44/16881; dated 12 June 2023). Additional institutional approvals were obtained from participating healthcare facilities, including King Abdulaziz Specialist Hospital, Taif (Approval No. HAO-02-T-105-44-099; February 2023), Alhada Armed Forces Hospital, Taif (Reference No. REC-2022-694; dated 27 December 2022), King Faisal Hospital, Taif (Approval No. 2023-B-24; dated 9 April 2023), and King Fahad Specialist Hospital, Dammam (Approval No. PHA0332; dated 28 March 2023). Participation was voluntary, and informed consent was obtained from all participants or caregivers prior to data collection. The study used a self-administered questionnaire, and no personally identifiable information (e.g., names, national identification numbers, addresses, or contact details) was collected. Survey responses were anonymous and analyzed in de-identified form. Access to the study data was restricted to the research team, and all data were handled confidentially in accordance with institutional ethical standards and the Declaration of Helsinki.

## 3. Results

### 3.1. Participant Characteristics

A total of 586 individuals completed the questionnaire, of whom 52 were excluded for not meeting the eligibility criteria, resulting in a final analytical sample of 534 participants. The largest proportion of participants were aged 25–44 years (44.0%), followed by those aged 18–24 years (26.6%). Females constituted slightly more than half of the sample (51.3%). Geographically, most participants were from the Western region (51.5%), followed by the Central region (38.4%). Regarding educational attainment, 36.7% had completed higher education and 33.5% had completed high school education. Clinically, generalized epilepsy was slightly more common than focal epilepsy (51.8% vs. 48.2%). Nearly half of participants (46.4%) reported experiencing a seizure within the preceding six months. A family history of epilepsy was reported by 21.0% of participants. Detailed sociodemographic and clinical characteristics are presented in Table 1.

### 3.2. Treatment Characteristics and Antiepileptic Medication Use

Treatment characteristics and patterns of AED use are presented in Table 2. Most participants were receiving monotherapy (60.86%), while 39.14% were on polytherapy. More than half of participants had been on AED treatment for longer than five years (55.62%), whereas 35.58% had been treated for 1–5 years. Levetiracetam was the most reported medication, used by 288 participants (53.93%), followed by carbamazepine (28.28%) and valproate (23.22%).

### 3.3. Adherence Levels

**Figure 1:**
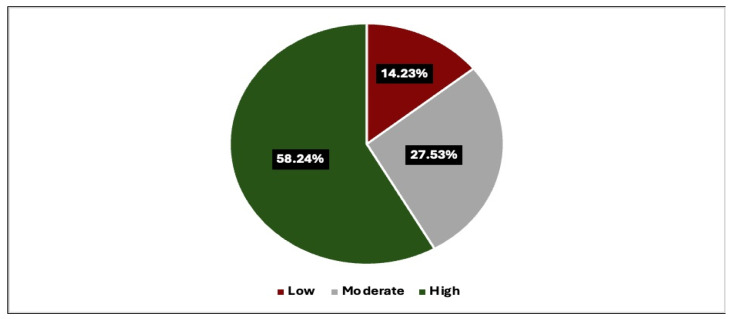
Distribution of medication adherence levels among participants (n = 534).

Among the 534 participants included in the analysis, more than half of the participants demonstrated high medication adherence. Specifically, 311 participants (58.24%) were classified as having high adherence (score 8–10), 147 (27.53%) had moderate adherence (score 6–7), and 76 (14.23%) exhibited low adherence (score 0–5). Overall, 41.76% of participants fell within the low-to-moderate adherence categories (Figure 1).

### 3.4. Adherence-Related Factors

The prevalence of self-reported factors potentially affecting medication adherence is presented in Table 3. The most reported factor was forgetting to take medication (57.3%), followed by fear of side effects (27.9%) and the high cost of medication (21.9%). Occurrence of medication side effects was reported by 20.4% of participants, while 19.1% cited a lack of health information. Other reported factors included fear of dependence (18.2%), stopping medication when feeling better (16.7%), feeling depressed (16.5%), and the belief that medication would not affect their condition (13.5%).

### 3.5. Reported Medication Side Effects

The prevalence of reported medication side effects is shown in Table 4. The most reported side effects were nervousness (35.6%), poor concentration (31.5%), headache (31.3%), fatigue (29.2%), and sleep problems (27.7%). Appetite changes (25.8%), tremor (24.5%), hair loss (21.9%), dizziness (22.9%), and gastrointestinal problems (22.5%) were also frequently reported.

### 3.6. Factors Associated with High Medication Adherence

In univariate logistic regression analysis, region, education level, a family history of epilepsy, seizure occurrence in the past six months, the number of adherence-related factors, and the number of side effects were significantly associated with high medication adherence. However, after adjustment for potential confounders in the multivariable model, a family history of epilepsy was no longer significantly associated with adherence (Table 5).

Compared with participants from the Southern region, those from the Eastern (aOR 5.06, 95% CI 1.14–22.35) and Central regions (aOR 5.36, 95% CI 1.48–19.38) had significantly higher odds of high adherence. Participants with no formal education were significantly more likely to report high adherence compared with those with higher education (aOR 3.22, 95% CI 1.29–8.04).

In contrast, patients who experienced seizures in the past six months had significantly lower odds of high adherence (aOR 0.39, 95% CI 0.26–0.59). Additionally, each incremental increase in the number of adherence-related factors (aOR 0.71, 95% CI 0.63–0.80) and reported side effects (aOR 0.92, 95% CI 0.87–0.97) was independently associated with reduced odds of high adherence.

## 4. Discussion

This study assessed the level of medication adherence among patients with epilepsy and identified demographic, clinical, and treatment-related factors associated with high adherence. More than half of participants demonstrated high medication adherence, although a substantial proportion reported moderate or low adherence. Several demographics, clinical, and treatment-related factors were independently associated with adherence. Regional differences emerged as significant predictors, with participants from certain regions demonstrating higher adherence compared with others. Educational level showed an unexpected pattern, whereby individuals with no formal education were more likely to report high adherence than those who had completed higher education. In contrast, recent seizure occurrence, a greater number of adherence-related barriers, and a higher burden of medication side effects were independently associated with lower odds of adherence.

The overall level of adherence observed in this study is comparable to reports from similar populations, where adherence rates among patients with epilepsy vary widely depending on measurement method and population characteristics [25,26]. Self-reported adherence tools often yield higher adherence estimates compared with pharmacy refill or electronic monitoring methods, which may partially explain the relatively high proportion of participants classified as highly adherent [27].

The finding that seizure occurrence was associated with lower adherence aligns with prior research suggesting a bidirectional relationship between seizure control and adherence [28,29]. Patients experiencing ongoing seizures may perceive treatment as ineffective, leading to reduced motivation to adhere. Conversely, poor adherence may contribute to inadequate seizure control [7,26]. Both explanations are plausible, and longitudinal studies are required to clarify the direction of this relationship.

The association between greater self-reported side effect burden and lower adherence is consistent with the broader epilepsy literature [30,31]. Although antiepileptic drug-related adverse effects have been associated with intentional and unintentional non-adherence [32], the effect observed in the present study was modest and should be interpreted with caution. Furthermore, because of the cross-sectional design, a temporal relationship cannot be established, and the reported symptoms may not be attributable solely to antiepileptic medications. Further longitudinal studies are needed to clarify the clinical significance and direction of this association.

Interestingly, individuals with no formal education demonstrated higher medication adherence compared with those with higher education. This finding contrasts with much of the literature, where higher educational attainment is often associated with better health literacy and improved adherence [33,34]. Therefore, this finding should be interpreted cautiously. One possible explanation is greater reliance on physician guidance and a stronger preference for physician-directed decision-making among individuals with lower educational attainment [35,36]. Alternatively, this pattern may reflect differences in health beliefs and expectations about treatment, as patients’ beliefs about medicines are known to significantly influence adherence behaviours [37,38]. It is also important to consider that self-reported adherence measures are susceptible to social desirability bias, which may lead to the overreporting of adherence, particularly in certain demographic groups [39,40]. Given the cross-sectional design of this study, causal relationships cannot be inferred. Further qualitative and prospective research would be valuable to confirm and better understand this unexpected relationship.

Regional variation in adherence suggests potential differences in healthcare access, service delivery models, socioeconomic factors, or patient education across regions [41,42]. These findings highlight the importance of contextual and system-level factors in influencing medication-taking behaviours [14]. The cumulative number of adherence-related barriers was a strong independent predictor of reduced adherence. This reinforces the multifactorial nature of adherence and supports the need for comprehensive assessment rather than focusing on a single determinant [14,43].

These findings have several important implications. Routine assessment of adherence-related barriers and medication side effects should be integrated into clinical practice. Clinicians should proactively inquire about cognitive, emotional, and financial challenges that may interfere with consistent medication use [14,44]. Additionally, patients experiencing recent seizures may require targeted adherence counselling and closer follow-up. Interventions should focus not only on dose adjustments but also on reinforcing the importance of consistent medication-taking behaviour. Moreover, the unexpected association between educational level and adherence suggests that adherence counselling should not be based solely on assumptions about health literacy. Individualized patient engagement strategies are essential. System-level differences reflected in regional variation indicate that adherence improvement strategies may need to be tailored to local healthcare infrastructure and patient populations [14].

### 4.1. Implications for Future Research

Future longitudinal studies are needed to clarify the temporal relationship between seizure occurrence and adherence. Objective adherence measures, such as pharmacy refill data or electronic monitoring, would strengthen future investigations and reduce potential reporting bias [44,45]. Qualitative studies exploring patients’ perceptions of medication effectiveness, side effects, and trust in healthcare providers may provide deeper insight into the behavioural drivers identified in this study. Additionally, intervention studies evaluating structured adherence support programmes in epilepsy populations are warranted.

### 4.2. Strengths and Limitations

This study has several strengths. It employed a multicentre design, recruiting participants from neurology outpatient clinics across multiple secondary and tertiary healthcare institutions in different regions of Saudi Arabia. This enhances the diversity of the sample and improves the generalisability of the findings within similar healthcare settings. In addition, this study comprehensively assessed demographic, clinical, behavioural, and treatment-related variables, allowing for a multidimensional evaluation of factors associated with medication adherence. Finally, the use of multivariable logistic regression enabled adjustment for potential confounding variables, strengthening the validity of the independent associations identified.

However, several limitations should be acknowledged. The cross-sectional design precludes causal inference, and the directionality of associations cannot be established. Adherence was assessed using the self-reported MARS, which was originally developed for patients with psychosis but has subsequently been widely used in studies of medication adherence across various chronic diseases, including epilepsy. As with other self-reported adherence measures, it may be subject to recall bias and social desirability bias. Additionally, recruitment using voluntary questionnaire participation may have introduced selection bias, as patients who regularly attend follow-up appointments and agreed to participate may have been more adherent than those who did not participate. Consequently, the prevalence of non-adherence may have been underestimated. Some subgroup analyses, particularly those involving participants from less represented geographic regions, included small numbers of participants, potentially resulting in unstable estimates and wider confidence intervals. Additionally, unmeasured variables such as healthcare accessibility, physician–patient communication quality, and medication availability may have influenced adherence but were not assessed in this study.

## 5. Conclusions

In this multicenter study of patients with epilepsy in Saudi Arabia, medication adherence was associated with regional, educational, clinical, and treatment-related factors. Seizure occurrence, greater adherence-related barriers, and medication side effects were associated with lower adherence. These findings may help inform targeted interventions to improve adherence among patients with epilepsy. However, given the cross-sectional design, causal relationships cannot be inferred, and prospective studies are warranted to confirm these associations.

## Figures and Tables

**Table 1:** Sociodemographic and clinical characteristics of participants (n = 534).

Characteristics		N (%)
*Age Group (years)*		
	16–17	49 (9.18%)
	18–24	142 (26.59%)
	25–44	235 (44.01%)
	45–59	63 (11.80%)
	≥60	45 (8.43%)
*Sex*		
	Female	274 (51.31%)
	Male	260 (48.69%)
*Region of residence*		
	Western region	275 (51.5%)
	Central region	205 (38.39%)
	Eastern region	30 (5.62%)
	Southern region	19 (3.56%)
	Northern region	5 (0.94%)
*Educational Level*		
	No formal education	43 (8.05%)
	Primary or intermediate education	116 (21.72%)
	High school	179 (33.52%)
	Higher education	196 (36.7%)
*Family History of Epilepsy*		
	No	422 (79.03%)
	Yes	112 (20.97%)
Type of Epilepsy *		
	Focal epilepsy	257 (48.22%)
	Generalized epilepsy	276 (51.78%)
*Seizure Attack in Last 6 Months*		
	No	286 (53.56%)
	Yes	248 (46.44%)

* One participant had missing data for type of epilepsy; percentages were calculated based on available data (n = 533).

**Table 2:** Treatment characteristics and current antiepileptic medication use among participants.

Characteristics		n (%)
*Type of Treatment*		
	Monotherapy	325 (60.86)
	Polytherapy	209 (39.14)
*Duration of Treatment*		
	<1 year	47 (8.8)
	1–5 years	190 (35.58)
	>5 years	297 (55.62)
Medication *		
	Levetiracetam	288 (53.93)
	Carbamazepine	151 (28.28)
	Valproate	124 (23.22)
	Lamotrigine	95 (17.79)
	Topiramate	38 (7.12)
	Other	32 (6.00)
	Lacosamide	23 (4.68)
	Gabapentin	17 (3.18)
	Phenytoin	16 (3.00)
	Clonazepam	15 (2.91)

* Percentages for medication use may exceed 100% as some participants were receiving combination therapy.

**Table 3:** Self-reported factors potentially affecting medication adherence among study participants.

Factor	n (%)
Forgetfulness in taking medication	306 (57.30)
Fear of medication side effects	149 (27.90)
High medication cost	117 (21.91)
Experiencing side effects	109 (20.41)
Inadequate health information	102 (19.10)
Fear of dependence on medication	97 (18.16)
Discontinuing medication when symptoms improve	89 (16.67)
Depressive symptoms	88 (16.48)
Perceived lack of medication effectiveness	72 (13.48)

**Table 4:** Self-reported antiepileptic drug-related side effects among study participants.

Side Effect	n (%)
Nervous	190 (35.58)
Poor concentration	168 (31.46)
Headache	167 (31.27)
Fatigue	156 (29.21)
Sleep problems	148 (27.72)
Appetite changes	138 (25.84)
Tremor	131 (24.53)
Hair loss	117 (21.91%)
Dizziness	122 (22.85)
Gastrointestinal problems	120 (22.47)
Pain in the bones	120 (22.47)
Depression	115 (21.54)
Confusion	107 (20.04)
Numbness	91 (17.04)
Aggressive behaviour	80 (14.98)
Poor sight	75 (14.04)
Allergy	55 (10.30)

**Table 5:** Univariate and multivariate logistic regression analyses of factors associated with high medication adherence.

Category		High Adherence %	OR (95% CI)	aOR (95% CI)
*Region of residence*				
	Southern	21.1%	1 (reference)	1 (reference)
	Eastern	60.0%	5.63 (1.50–21.12)	5.06 (1.14–22.35)
	Northern	40.0%	2.50 (0.31–20.45)	4.21 (0.41–43.05)
	Western	49.8%	3.72 (1.21–11.50)	2.73 (0.78–9.49)
	Central	73.2%	10.23 (3.25–32.15)	5.36 (1.48–19.38)
*Education*				
	Higher education	55.1%	1 (reference)	1 (reference)
	No formal education	81.4%	3.56 (1.57–8.08)	3.22 (1.29–8.04)
	Primary/intermediate	57.8%	1.11 (0.70–1.77)	0.81 (0.46–1.40)
	High school	56.4%	1.06 (0.70–1.59)	0.74 (0.46–1.21)
*Family History*				
	No	61.6%	1 (reference)	1 (reference)
	Yes	45.5%	0.52 (0.34–0.79)	0.97 (0.58–1.61)
*Seizure (last 6 months)*				
	No	70.3%	1 (reference)	1 (reference)
	Yes	44.4%	0.34 (0.24–0.48)	0.39 (0.26–0.59)
Number of factors		—	0.63 (0.56–0.70)	0.71 (0.63–0.80)
Number of side effects		—	0.85 (0.81–0.89)	0.92 (0.87–0.97)

High adherence defined as adherence score 8–10. OR = crude odds ratio; aOR = adjusted odds ratio; CI = confidence interval. Reference categories are indicated as 1 (reference). Model fit: likelihood ratio χ^2^(12) = 156.53 (*p* < 0.001); pseudo R^2^ = 0.216.

## Data Availability

The datasets used and analyzed during the current study are available from the corresponding author on reasonable request due to data privacy considerations.

## Supplementary Materials

The following supporting information can be downloaded at: https://www.mdpi.com/article/10.3390/healthcare14152302/s1, Table S1: Distribution of participants by recruiting centre, city, and region of residence.
