# Supplementary material for: Antiepileptic Drug Adherence in Patients with Epilepsy in Saudi Arabia: A Multicentre Cross-Sectional Study
Source: Healthcare (Basel). 2026 Jul 30;14(15):2302. doi: 10.3390/healthcare14152302 (PMC13465262; doi:10.3390/healthcare14152302)
Supplement: Supplementary file 1 [file healthcare-14-02302-s001.zip › healthcare-4411226-supplementary.pdf]

Table S1: Distribution of participants by recruiting centre, city, and region of residence.

Participating hospital	City	Participants recruited, n (%)	Western, n (%)	Central, n (%)	Eastern, n (%)	Northern, n (%)	Southern, n (%)
**Hospital 1**	AL Qassim	192 (36.0)	9 (4.7)	180 (93.8)	0 (0.0)	2 (1.0)	1 (0.5)
**Hospital 2**	Taif	86 (16.1)	82 (95.3)	0 (0.0)	0 (0.0)	0 (0.0)	4 (4.7)
**Hospital 3**	Taif	180 (33.7)	172 (95.6)	1 (0.6)	1 (0.6)	2 (1.1)	4 (2.2)
**Hospital 4**	Dammam	54 (10.1)	0 (0.0)	24 (44.4)	29 (53.7)	1 (1.9)	0 (0.0)
**Hospital 5**	Jeddah	22 (4.1)	12 (54.5)	0 (0.0)	0 (0.0)	0 (0.0)	10 (45.5)

Note: Percentages in the *Participants recruited* column are based on the total study population (N = 534), whereas percentages in the *Region of residence* columns are calculated within each participating centre.
